# MALDI-TOF MS as a Novel Tool for the Estimation of Postmortem Interval in Liver Tissue Samples

**DOI:** 10.1038/s41598-017-05216-0

**Published:** 2017-07-07

**Authors:** Chengzhi Li, Zhengdong Li, Ya Tuo, Dong Ma, Yan Shi, Qinghua Zhang, Xianyi Zhuo, Kaifei Deng, Yijiu Chen, Zhenyuan Wang, Ping Huang

**Affiliations:** 1 School of Forensic Science and Medicine, Xi’an Jiaotong University, Xi’an, 710061 China; 2 Shanghai Key Laboratory of Forensic Medicine, Shanghai Forensic Service Platform, Institute of Forensic Science, Ministry of Justice, P.R.China, Shanghai, 200063 China; 3 Department of Biochemistry and Physiology, Shanghai University of Medicine and Health Sciences, Shanghai, China; 4 Shanghai Key Laboratory of Forensic Science, Institute of Forensic Science, Ministry of Justice, Shanghai, 200063 China

## Abstract

Estimation of the postmortem interval (PMI) is a complicated task in forensic medicine, especially during homicide and unwitnessed death investigations. Many biological, chemical, and physical indicators can be used to determine the postmortem interval, but most are not accurate. Here, we present a novel matrix-assisted laser desorption/ionization time-of-flight mass spectrometry (MALDI-TOF MS) method that can be used for the estimation of PMI using molecular images and multivariate analyses. In this study, we demonstrate that both rat and human liver tissues of various PMIs (0, 2, 4, and 6days) can be discriminated using MALDI imaging and principal component analysis (PCA). Using genetic algorithm (GA), supervised neural network (SNN), and quick classifier (QC) methods, we built 6 classification models, which showed high recognition capability and good cross-validation. The histological changes in all the samples at different time points were also consistent with the changes seen in MALDI imaging. Our work suggests that MALDI-TOF MS, along with multivariate analysis, can be used to determine intermediate PMIs.

## Introduction

Postmortem interval (PMI) estimation is an important task in daily forensic casework.

The physical changes that occur after death, including the cooling of the body, rigor mortis, and the development of lividity, have long been recognized as early postmortem phenomena^1^. These signs continue to be the main basis for estimating the PMI^1^. For many years, various approaches have been used to determine the PMI, including examinations of thanatochemistry^2, 3^, DNA/RNA degradation^4, 5^, and forensic entomology^6, 7^. However, these methods estimate the postmortem interval using a few—or even just one—specific parameters, and their precision and time-frame of applicability are often limited. In recent years, studies of postmortem microbial communities^8–11^, and postmortem metabolomics/lipidomics^1, 12, 13^, have emerged, and have been successfully applied to PMI estimation. These technologies may be a potential tool for PMI estimation in forensic practice.

Since its introduction in 1985 by Hillenkamp *et al*.^14^, matrix-assisted laser desorption ionization (MALDI) has seen rapid development in the life sciences and medicine^15^. An important component of the success of this technique has been MALDI imaging mass spectrometry (IMS) of tissue sections, which was introduced in 1997 by Caprioli and coworkers^16^. The label-free nature and lack of requirement for prior knowledge make it highly suitable for both explorative, as well as comparative, research of various tissue samples^17^. The use of MALDI IMS offers the ability to investigate pathophysiological changes taking place directly in a tissue while retaining the histopathological context, allowing the simultaneous mapping of hundreds of peptides and proteins present in tissue sections, with a lateral resolution of approximately 50–75 microns^18^. MALDI IMS is evolving into a powerful tool for biomedical research^19^, and this technology has been applied to a variety of analyte classes, including pharmaceuticals^20, 21^, metabolites^22^, lipids^23^, peptides^24, 25^, and proteins^26, 27^.

When a rat or human dies, the internal organs will decay in a time-dependent manner. Therefore, the relative intensities of signals from peptides or proteins will vary by body organ and postmortem interval. To address the effect of the time since death on the decomposition of internal organs, we describe here results of a study on postmortem rat and human liver tissues. We hypothesized that, as a rat or human body decomposes, the relative signal intensities from different peptides or proteins within the internal organs will change. To assess this hypothesis, we measured rat and human liver samples at various time intervals after death (range = 0–144 hours) using MALDI-TOF MS on peptides and proteins, and used a profiling acquisition, imaging, and bioinformatics package for inspection and comparison of data sets.

## Results

### Observation of changes in rat and human liver samples at different PMIs with HE staining

**Figure 1:**
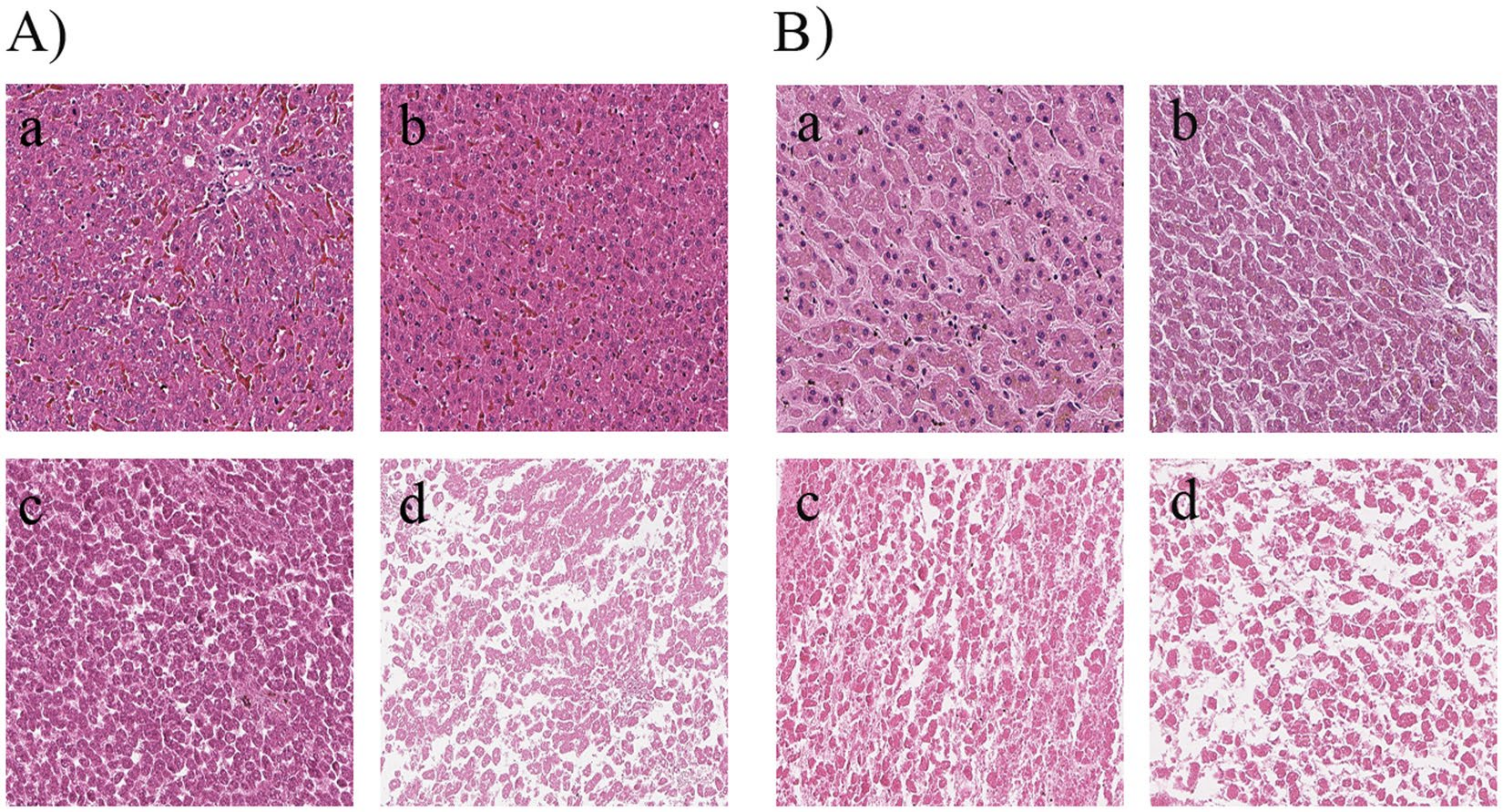
Histological changes at (a) 0, (b) 48, (c) 96, and (d) 144 h postmortem for liver tissues. (**A**) in SD rat liver samples; (**B**) in human liver samples.

From a PMI of 0 h to that of 144 h, the severity of both rat and human liver cell autolysis continuously increased, as shown in the micrographs of postmortem liver cells (hematoxylin and eosin staining, 20× magnification) (Fig. 1). At 0 h, all of the liver cells and cell nuclei were clearly visible. At 48 hours after death, the nuclei were observed to shrink, and partial degradation was seen in the hepatocytes (Fig. 1). After 96 hours, the nuclei completely disappeared, and 144 hours after death, the liver cell structure itself had mostly disappeared.

### MALDI imaging of liver samples from rats and humans

**Figure 2:**
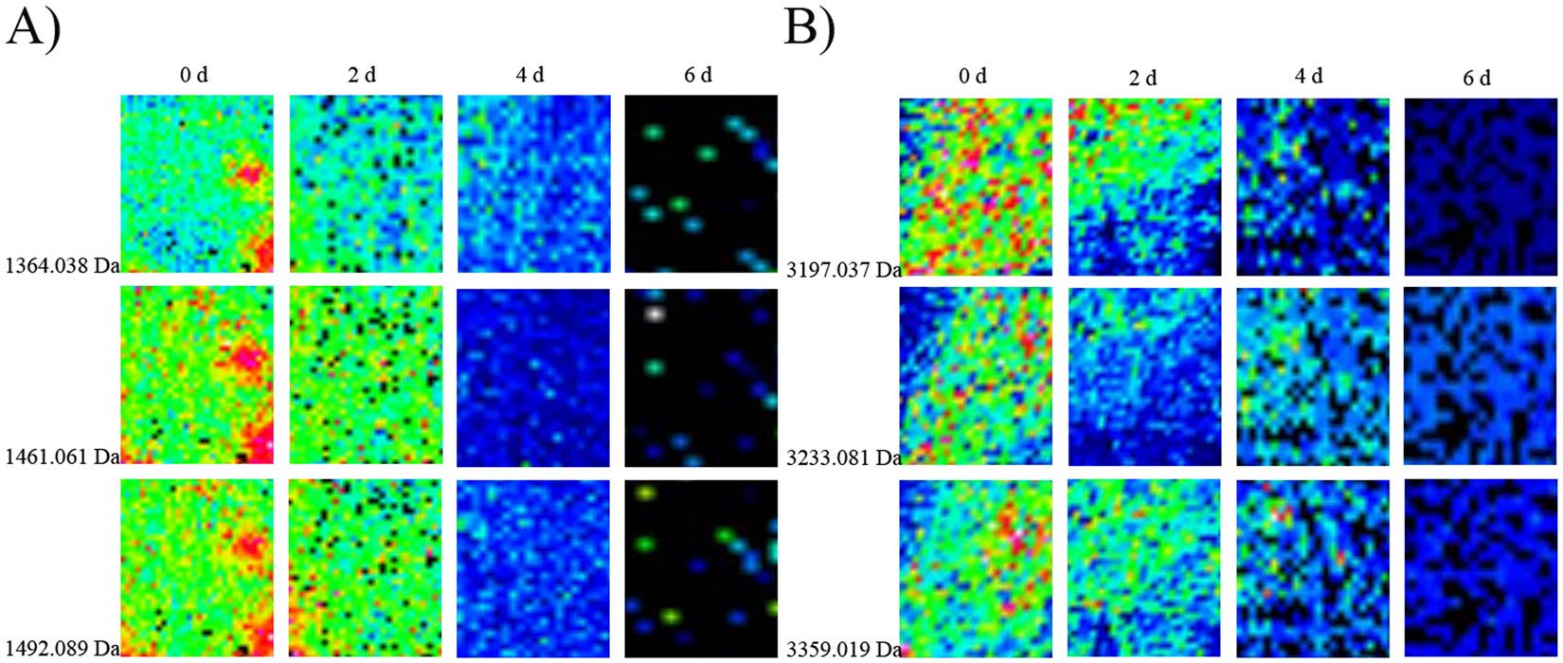
Ion signals showing an obvious change at different PMIs. (**A**) In SD rat liver samples; (**B**) in human liver samples.

**Figure 3:**
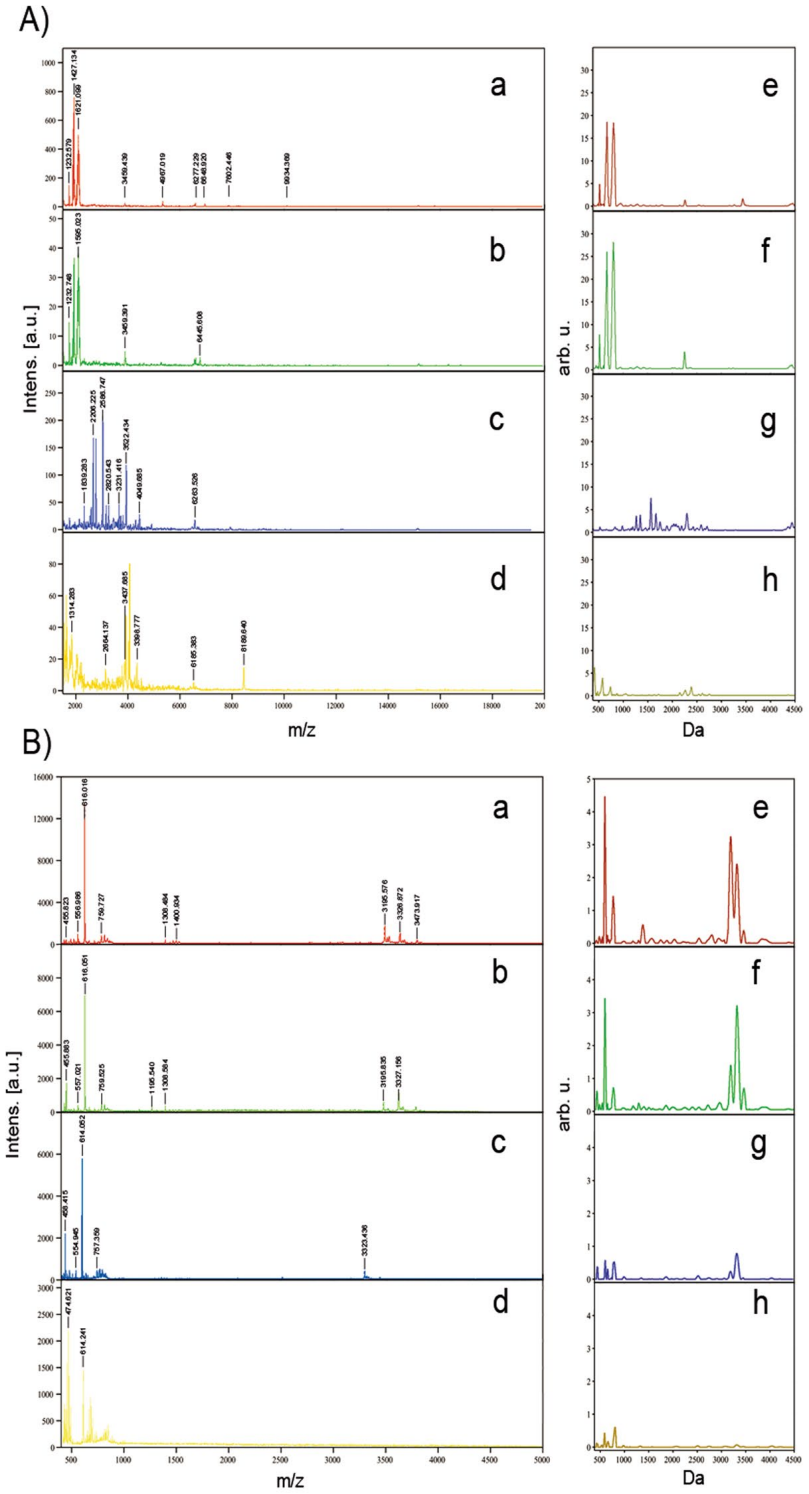
(a–d) Representative mass spectrum at (a) 0, (b) 48, (c) 96, and (d) 144 h postmortem for liver tissues. (e–h) Average peptide/protein profiles at (e) 0, (f) 48, (g) 96, and (h) 144 h postmortem for liver tissues. (**A**) In SD rat liver samples; (**B**) in human liver samples.

All of the liver tissue sections, obtained from different PMI groups, were examined using hematoxylin and eosin staining, as well as MALDI imaging. After acquiring imaging data from all the tissue sections, the FlexImaging 3.0 software program was used to select peptides with strong spectral signals to generate MALDI images (Fig. 2) for the different PMI groups. From the spectra, we found that most peaks were in the 700–4000 Da range (Fig. 3). Before the generation of ion density maps, the spectra were normalized to the total ion current to minimize spectrum-to-spectrum differences in peak intensities. For the generated images, the peaks at *m*/*z* = 1364.038, 1461.061, and 1492.089 in the rat liver tissues, and 3197.037, 3233.081, and 3359.019 in the human liver tissues, for example, showed marked decreases in intensity between 0 h and 144 h postmortem (Fig. 2).

### Processing MALDI IMS data using PCA

**Figure 4:**
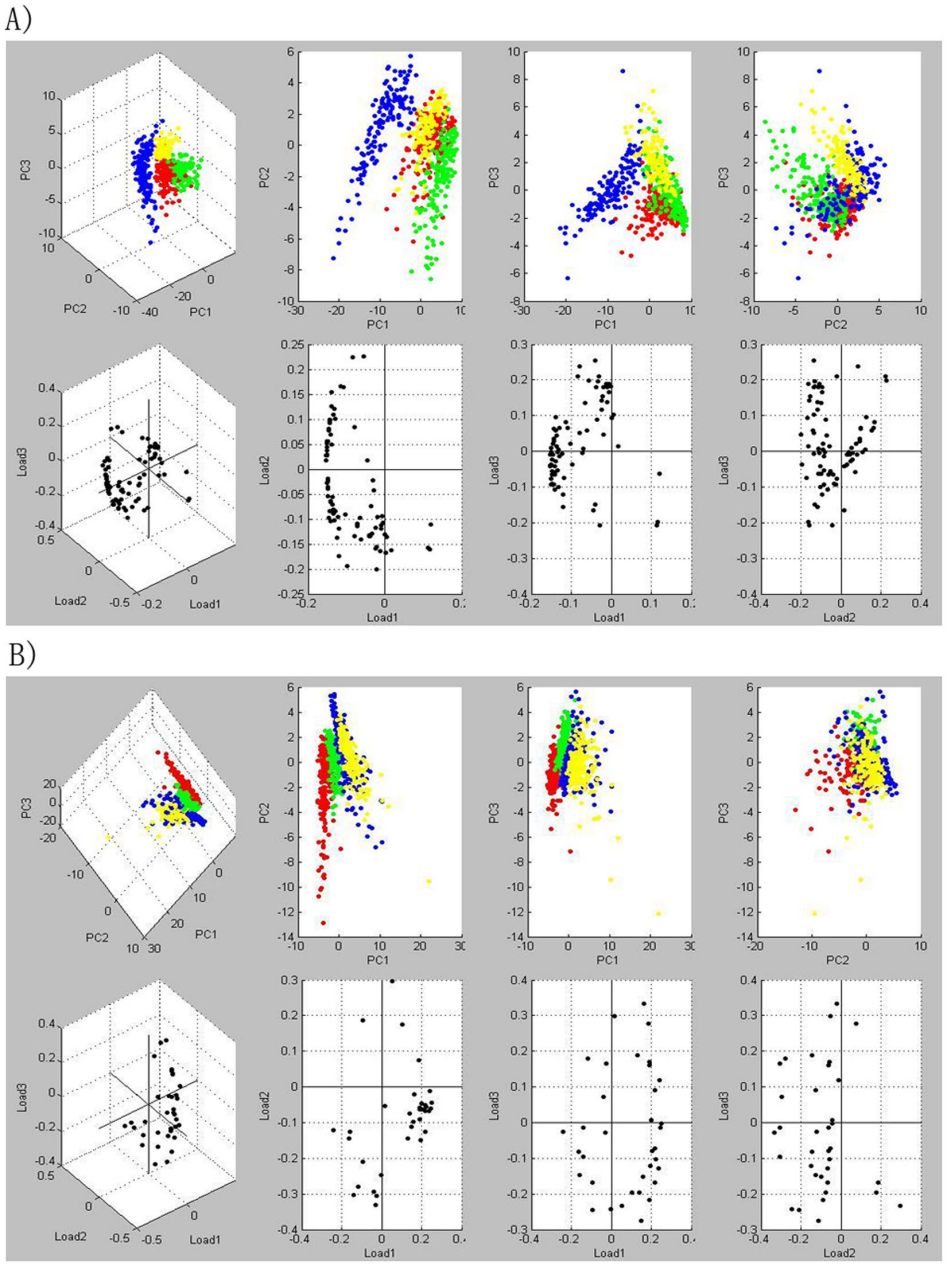
Principal component analysis (PCA) of MALDI IMS data acquired in the liver samples at 0 h (red), 48 h (green), 96 h (blue), 144 h (yellow) postmortem. (**A**) In SD rat liver samples; (**B**) in human liver samples.

Multiple spectra (*n* ≈ 150) per region of interest were selected from the MALDI IMS data. Comparisons of regions of interest for the different PMI groups were conducted with a principal component analysis (PCA) based on peptide or protein patterns. Using PCA, the different PMI groups were separated from each other successfully (Fig. 4). This means that the various peptides and proteins present in the rat and human liver samples change significantly with time after death.

### Gel view of PMI group data

**Figure 5:**
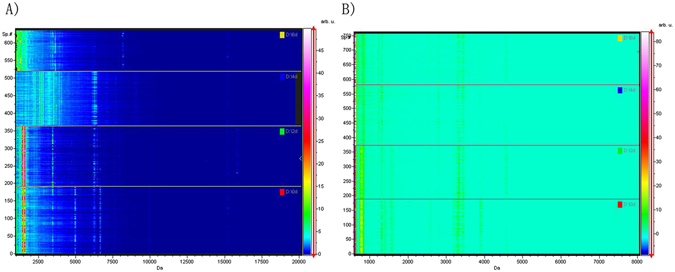
Gel view of data from different PMI groups. (**A**) In SD rat liver samples; (**B**) in human liver samples.

The gel view displays all of the spectra from the loaded classes arranged into a pseudo-gel configuration. The x-axis records the m/z value, while the y-axis displays the running spectrum number originating from subsequent spectral loading. The peak intensity is expressed by a color code. The color bar and the y-axis indicate the relationship between the color of a peak and the corresponding intensity in arbitrary units. The intensities of the major peaks for each PMI group were significantly different from those of all other PMI groups (Fig. 5).

### Differences in peaks at various PMIs and establishment of classification models

**Figure 6:**
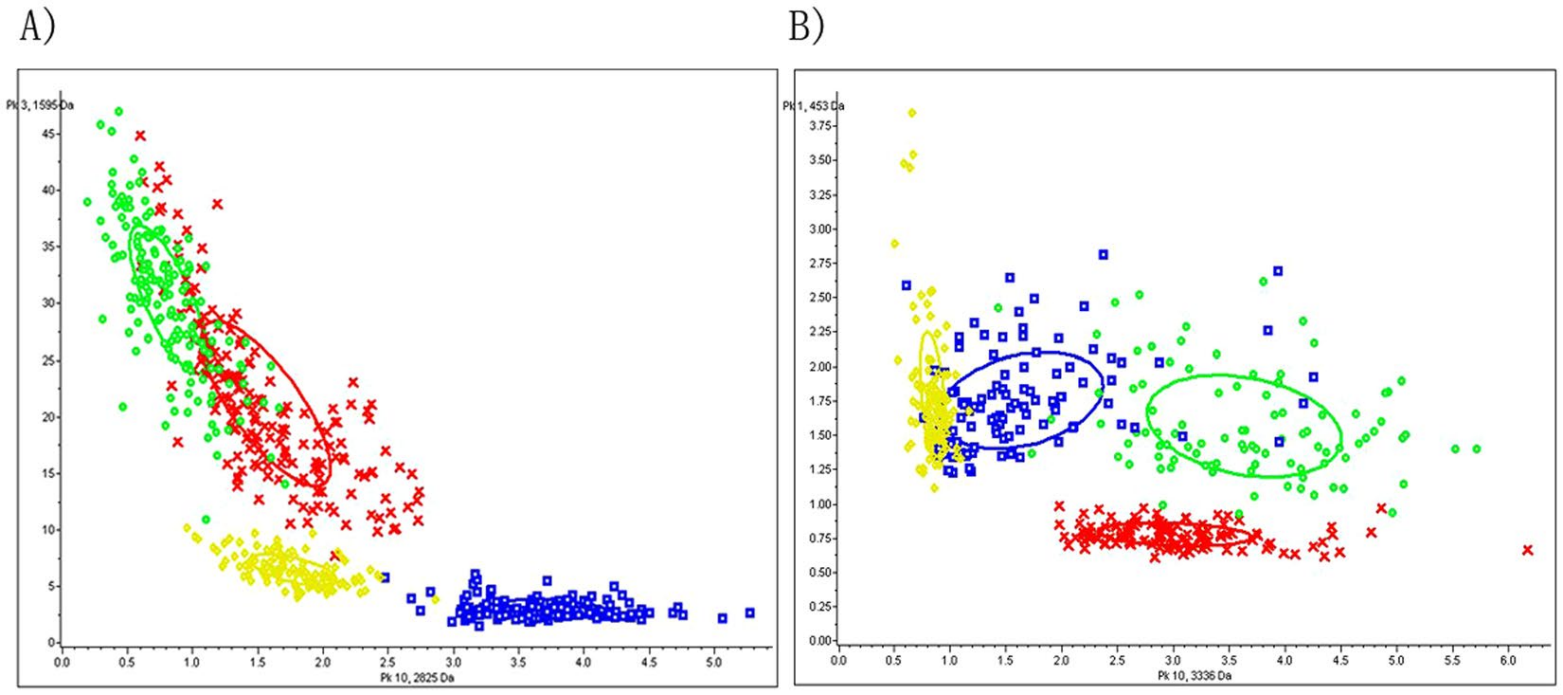
Two-dimensional peak distributions of peptides with *m*/*z* = 2825.524, 1595.481, 3335.815, and 453.348 for different PMI groups. (**A**) In SD rat liver samples; (**B**) in human liver samples.

Overall, 57 peptide/protein peaks from rat liver samples, and 43 from human liver samples, showed significant differences in the spectra of the training group data sets generated by MALDI-TOF MS (Supplementary Tables S1 and S2). Peptide/protein peaks with m/z = 2825.524 and 1595.481 in rat liver tissues, and 3335.815 and 453.348 in human liver tissues, exhibited the greatest differences in peak intensity between different PMIs (p < 0.00001). Because of this, these peaks were plotted as 2D peak distributions (Fig. 6). Three algorithms, GA (optimized by adjusting the number of neighbors for k-nearest neighbor classification), SNN, and QC, were used for classification model construction using spectral data from the training group generated by MALDI-TOF MS. The recognition capability, cross-validation, and overall accuracy of the models are presented in Tables 1 and 2.

**Table 1:** Cross-validation and recognition capability of the three algorithms used to classify different PMIs in rat liver tissues.

Algorithm	Model name	Cross-validation (%)	Recognition capability (%)	Overall accuracy (%)
GA	GA	92.16	95.50	92.20
SNN	SNN	74.87	96.38	83.34
QC	QC	79.85	79.53	75

**Table 2:** Cross-validation and recognition capability of the three algorithms used to classify different PMIs in human liver tissues.

Algorithm	Model name	Cross-validation (%)	Recognition capability (%)	Overall accuracy (%)
GA	GA	80.96	91.07	90.73
SNN	SNN	88.12	95.54	92.40
QC	QC	82.59	85.71	82.60

## Discussion

Precise estimation of the PMI has been difficult since the very beginning of forensic medicine. It is one of the fundamental tasks of the forensic pathologist summoned to the scene where a body has been found^28^. MALDI IMS is an effective tool that provides molecular images of tissues during the molecular discovery process^29^. It has high speed and throughput, simple sample handling, and excellent reproducibility^30^. This powerful technology is widely used for proteome and peptidome imaging. By collecting mass spectra at discrete spatial points, one can generate ion images reflecting the localization of biomolecules. With this information, one can further map and study the 2D molecular profile of a tissue section. These benefits make it an ideal method for the estimation of PMI. The liver is optimal for this process because it exhibits autolysis, rapid putrefaction, and the presence of a homogenous cell population, which alleviates the effect of between-cell variation^31^.

MALDI IMS is being used increasingly often for disease diagnosis and classification^32, 33^. In this study, we investigated the use of MALDI IMS for profiling the proteins and peptides in rat and human liver tissue samples of different PMIs. The peaks at *m*/*z* = 1364.038, 1461.061, and 1492.089, for example, in rat liver tissues, and 3197.037, 3233.081, and 3359.019 in human liver tissues, strongly decreased in intensity with increasing PMI (Fig. 2). These changes detected with MALDI IMS are consistent with the histological changes in rat and human liver tissues with intermediate PMIs Fig. 1. In our previous study^31^, we also found the infrared microspectral imaging changes were consistent with pathological postmortem changes in rat liver tissues.

The intensities of peptide/protein peaks, such as *m*/*z* 1595.481, 2825.524, 1426.773, 3810.226, 4621.661 in rat liver tissues and 453.348, 3335.815, 3208.154, 616.574, 3476.852 in human liver tissues, changed significantly from time zero to 144 h postmortem. In total, four protein signals in rat liver tissues, and three in human liver tissues were identified (Tables 3 and 4). At 48 h postmortem, some new peaks appeared both in rat and human liver tissues, suggesting that the liver peptide/protein degraded obviously on account of autolysis or decomposition. At 96 h postmortem, some peaks disappeared gradually, suggesting that the liver peptide/protein degraded seriously. After 144 h postmortem, few or no peaks can be detected by MALDI-TOF MS system, suggesting that the liver peptide/protein degraded completely. Our results showed that the significantly changed peptide/protein peaks in rat liver tissues were not the same as in human liver tissues. That may be result from the difference of internal environments, such as enzymes activity and microbial activities, in rat and human livers. Though the constructed PMI classification models with a high recognition capacity and good cross-validation in both the rat and human liver tissues, the model built in rats can’t be used to predict PMI in humans. Overall, the postmortem changes in rat liver tissues were similar with human liver tissues.

**Table 3:** The identification of the potential markers used for the estimation of PMI in rat liver tissues.

Protein	Meas. M/z	Calc. MH^+^	Number of matched peptides	Mascot Score	Sequence
uncharacterized protein LOC102151723	9934.369	9939.046	6	56.10	TWAVVSDAVGCVEGALRPVAQVGQHQAPVTQVGQHQAPLTQITMSVYTVAALPGPWGCSRDSTTACSALAPWPSPSLPTATLPAHGAQTVPLLGVHI
basic proline-rich protein-like	6274.792	6275.068	4	54.80	LLQADQHRAPSTPAPTADGAGGSAASPAHPEPQPIAGGGGGGGGGAGTSSPAAGARPGPPRPAPPPACR
olfactory receptor 2G3-like	6252.396	6258.314	7	63.20	ALGTCGSHLLVVSLFYGTITAVYIQPNSSYAHTHGKFISLFYTVVTPTLNPLIYTLR
interferon omega 5 precursor	3918.777	3916.990	6	59.30	TQAISVLHEMLQQTFLLFHTERSSAAWDSTLLDK

**Table 4:** The identification of the potential markers used for the estimation of PMI in human liver tissues.

Protein	Meas. M/z	Calc. MH^+^	Number of matched peptides	Mascot Score	Sequence
Rho GTPase-activating protein 24	3233.339	3233.619	4	44.20	MGILNSDTLGNPTNVRNMSWLPNGYVTLR
Amine oxidase	3327.452	3328.533	5	32.70	QPVDRIYFAGTETATHWSGYMEGAVEAGER
Small vasohibin-binding protein	686.286	686.329	1	14.50	MDPPAR

PCA is a common mathematical technique that is used for the analysis, visualization, and compression of biological data. The primary goal of PCA is to reduce the dimensionality of a data set, while simultaneously retaining the information present. This technique transforms the original variables, defined by peaks intensities, to new variables, which are called the principal components (PCs), which best explain the variance in the data set^18^. This variable transformation is defined in such a way that the first PC has the largest possible variance, with the ultimate goal of discarding the components that are not significant, while reconstructing images that contain most of the original information^18^.

In this study, MALDI imaging data for samples taken at PMIs of 0 h, 48 h, 96 h, and 144 h in both rat and human liver tissues were analyzed using PCA. The PCA score plots showed that the different groups were well separated from each other for both rat and human liver tissues Fig. 4. Respectively, PC1, PC2, and PC3 describe 36%, 15%, and 12% of the original observation variability in rat liver tissues, and 30%, 19% and 11% in human liver tissues. These were chosen to generate score plots (Fig. 4). The potential chemical markers that contributed most to the differences of the different time groups were observed from the loading plot. In this plot, ion signals at m/z = 2825.524, 1595.481, 1426.773, 3810.226, and 3905.093 in rat liver tissue, and 790.992, 453.348, 3476.852, 3335.815, and 616.574 in human liver tissue were selected as potential markers for the estimation of PMI. Generally, the intensity of these ion signals exhibited large differences among the different PMIs.

In our study, the GA, SNN, and QC algorithms were used to build classification models based on the MALDI IMS data set derived from the different PMI groups. The classification of different time group data yielded high recognition capacity and good cross-validation in both the rat and human liver tissues (Tables 1 and 2). The application of our model to different test sets also achieved high accuracy in discriminating PMI groups. In short, we generated classification models both in rat and human liver tissues based on MALDI IMS-derived spectral data, and these models were well suited for the accurate estimation of PMI.

Previous studies have shown notable trends in the relative abundances of different bacterial taxa in the buccal cavities and rectums of rat corpses, as well as the internal organs, buccal cavities, and blood of human corpses throughout the decomposition process^9, 34^. Metcalf *et al*. also found that postmortem microbial communities changed in a clock-like manner that provided an estimate of absolute PMI, which is similar to the use of the development of fly larvae to estimate the PMI^10^. In this study, we found that the peptides or proteins of the rat and human livers also showed obvious changes at different PMIs. Takako Sato *et al*. detected 70 metabolites in cardiac blood and found that among these, 25 metabolites had a strong statistical correlation with the PMI, which was similar to the report of Donaldson *et al*.^1, 12^. Kaszynski *et al*. identified 175 and 163 metabolites in muscle and serum samples of mice, respectively, and found that 17 (9.7%) and 14 (8.5%) of the total number of metabolites demonstrated significant correlation with the PMI^35^. In our study, we detected 57 and 43 peaks in rat and human liver tissues, respectively, and found that these peaks had a strong statistical correlation with the PMI. In general, the results of this study in rat livers were in good agreement with our previous studies^31, 36^. To date, this study is the first to combine MALDI imaging mass spectrometry with multivariate analysis for the estimation of postmortem interval in both rat and human models.

Although MALDI IMS is a promising method for PMI estimation, it also has limitations. The sample preparation process must be carried out as quickly as possible without exposing the samples to air, moisture, or high temperatures. Also, all types of sample treatments, including cutting, washing, or matrix application, can lead to sample contamination and molecular diffusion, which could negatively affect the reproducibility of the data, complicate the analysis, or affect the quality of the image^37^. Additionally, in this work, we only studied rat and human samples at constant temperature. The effects of various factors, such as temperature, humidity, and cause of death, should be considered in future studies.

In conclusion, we demonstrated that MALDI IMS, combined with multivariate analysis, can be utilized to estimate intermediate PMIs based on protein or peptide signatures in rat and human liver tissue samples. Large correlations between MALDI IMS and histological changes were also found in both rat and human liver tissues. The classification models constructed can be used for the estimation of PMI under specific conditions.

## Materials and Methods

### Materials and instruments

Trifluoroacetic acid, acetonitrile, and ethanol were purchased from Sigma-Aldrich (St. Louis, MO). An Autoflex III SmartBeam MALDI-TOF MS system, MTP Slide Adapter II, ImagePrep, conductive slides, sinapinic acid, and α-cyano-4-hydroxy cinnamic acid were purchased from Bruker Daltonics (Bremen, Germany). Optimum cutting temperature (OCT) compound was obtained from Leia (Leica Microsystems Nussloch GmbH, Germany). A peptide calibration standard was also obtained from Bruker Daltonics, which included angiotensin II, angiotensin I, substance P, bombesin, adrenocorticotrophic hormone clip 1–17, adrenocorticotrophic hormone clip 18–39, somatostatin 28, and rennin substrate. A Labconco Centrivap cold trap and concentrator was obtained from LabconcoCorp. (Kansas City, MO, USA). A Leica CM 1950 cryostat was purchased from Leica (Leica Biosystems Nussloch GmbH, Germany).

### Sampling procedures

Thirty-six adult Sprague-Dawley rats weighing between 250 and 300 g, and 24 *in vitro* right posterior lobe of livers (human) weighing 200 g, were kept in an incubator under constant conditions (23 ± 1 °C, 30–45% relative humidity). Before euthanasia by mechanical asphyxia, the rats were sedated with subcutaneous injection of phenobarbital sodium (50–60 mg/kg). Rat liver samples were collected by dissection at four PMIs (0, 48, 96, and 144 h). *In vitro* right posterior lobe of livers (human) were obtained from individuals who suffered sudden cardiac death or car accident, and the corpses were kept in a freezer starting immediately after death (Supplementary Table S3). The time when a corpse was dissected was marked “0 h”. *In vitro* right posterior lobes of livers (human) were collected after dissection at four “PMIs” (0, 48, 96, and 144 h). For sampling, the right posterior lobe of livers (human) were cut into broad strips using two parallel razor blades mounted 8 mm apart, and the strips were subsequently trimmed to an average size of 10 mm × 10 mm × 8 mm. Then, the liver strips were washed with phosphate-buffered saline (PBS), loosely wrapped in aluminum foil, and frozen in liquid nitrogen at temperatures below −70 °C by gently lowering the tissue into liquid nitrogen and maintaining it for 30–60 s. Finally, all samples were stored in a freezer at −80 °C until they were required for analysis.

This study was approved by the Science and Ethics Committee of Xi’an Jiaotong University Health Science Center, and the methods were carried out in accordance with the approved guidelines and regulations. Informed consent was obtained from next-of-kin relatives of the cases.

### Sample preparation

For MALDI-TOF MS analysis, serial sections (10 µm) were cut from the frozen tissue samples with a Leica CM 1950 cryostat at −20 °C. The sections were placed with forceps onto either a regular glass slide for hematoxylin and eosin (HE) staining or an indium tin oxide-coated glass slide (Bruker Daltonics) for MALDI analysis. Prior to rinsing with 70% ethanol and 100% ethanol for 12 s, the sections for MALDI analysis were transferred to a cold room (−4 °C) and dried for 10 min in a Labconco Centrivap cold trap and concentrator. After washing with ethanol, the sample was dried again. Tissue fixation and removal of salts and other contaminants was carried out through a series of ethanol/water washing steps, as described in previous studies^37–40^. Later, the MALDI matrix was applied using the ImagePrep system, and the standard protocol provided with the instrument was followed. A solution of sinapinic acid (10 mg/mL) in water/acetonitrile 40:60 (*v*/*v*) with 0.2% trifluoroacetic acid was used as the matrix for the MALDI measurements of rat and human liver tissues. The Bruker default method consisted of 5 phases in total, each including individual cycles of incubation, nebulization and dehydration. Phases 2–5 were controlled by an optical sensor, which measured the moisture, matrix layer thickness, and dehydration parameters, by monitoring the scattered light intensity by refraction index matching^41^. The amount of matrix was then applied in a defined range (minimum/maximum number) of cycles based on a given sensor signal or the defined range of spray cycles that are specified within each phase^41^. Spray time was approximately 2 s, and incubation time was 30 s. The detailed pretreatments for peptide/protein analysis, such as fresh frozen tissue sample preparation and matrix application, were prepared as reported in earlier studies^37–42^.

### MALDI-TOF MS analysis

Analysis of tissue sections was performed using an Autoflex III SmartBeam MALDI-TOF mass spectrometer equipped with a 337-nm Nd:YAG laser. The spectra were recorded in the positive linear mode (delay: 200 ns; ion source 1 (IS1) voltage: 20.00 kV; ion source 2 (IS2) voltage: 18.90 kV; lens voltage: 6.5 kV; sampling rate, 0.1 GS/s; mass range: 400 Da to 10,000 Da; peak assignment tolerance: 20 ppm). For MALDI IMS data acquisitions, 500 shots were summed per array position with a spatial resolution of 1000 µm. Data acquisition was carried out at 44% of the maximum laser energy. Software used for data acquisition was Flex Control 3.0 (Bruker Daltonics) with the LP_ProtMix method,which is mainly used for peptides or proteins analysis. Prior to the analysis of a tissue sample on the plate, external calibration was accomplished to minimize the mass shift by using a calibration mixture of peptides and proteins, which was deposited on the surface of the sample support materials and covering the mass range of 700–4000 Da.

### Statistical analysis and spectral classification

The ClinProTools software 2.2 (Bruker Daltonics) was used for the analysis of all imaging data derived from the sections of the rat and human liver samples taken at different PMIs. All data comparisons were performed equally, summarizing signals above a signal-to-noise ratio greater than 3.0. All spectra were routinely baseline-corrected using the Top Hat algorithm with a 10.0% minimal baseline width, and smoothed using the Savitzky–Golay algorithm with a 5.0 width (m/z) and 2 cycles. The intensities of the peaks of interest were normalized with the peak intensity of an ACTH internal standard. The pretreated data were then used for visualization and statistical analysis using ClinProTools.

Differences in peptide peaks among the different time groups were selected using the peak intensity as the basis of statistical differences. Built-in mathematical models in ClinProTools2.2 (the GA, SNN algorithm, and QC algorithm) were then used to select peptide peaks and set up classification models to determine the optimal separation planes between samples from different PMIs.

After each model was generated, a random cross-validation process was carried out with the software, and the percent to leave out and number of iterations were set at 20 and 10, respectively. The models were then validated using the test set in a blinded manner. To determine the accuracy of the class prediction model, the software quantifies recognition capability and cross-validation. Recognition capability describes the performance of an algorithm, i.e., the proper classification of a given data set^43^. Cross-validation is a measure of the reliability of a model and can be used to predict how a model will behave in the future. This method is used for evaluating the performance of an algorithm for a given data set and under a given parameterization^43^. To minimize sampling errors, the selection for the training and test sets was repeated 30 times. The classification results given in the tables are the average of the 30 single classification results. For multivariate analyses, PCA was performed to observe the differential distribution of masses at the surfaces of the tissues.

### Peptide/protein Identity Assignment

MALDI MS spectra of each selected peptide were obtained using an Autoflex III SmartBeam MALDI-TOF MS instrument operated in linear positive ion mode with spectra acquired in the range of m/z 400–10,000. The obtained spectra were processed using flexAnalysis 3.3 (Bruker Daltonik). Data were submitted to Mascot Server 2.5.14 (Matrix Science, Boston, USA) and run against the SwissProt database (SwissProt 2016_10) in human sample and NCBIprot database (NCBIprot_20161127) in rat sample to match the peptide sequences to their corresponding intact proteins. Trypsin was chosen as the protease in the search parameter. SwissProt database search included 552884 sequences, and 197760918 residues; specifically from the taxonomy “Homo sapiens” 20121 entries were searched. NCBIprot database search included 106762850 sequences, and 39119668168 residues; specifically from the taxonomy “Other mammalia” 1787181 entries were searched. The protein N-term, was selected for modification in human samples, and carbamidomethyl (C) for rat samples, respectively (Tables 3 and 4).

### Application of the complete classification models

#### Cadaver cases and sample collection

Liver samples derived from four human corpses (2 males and 2 females) from routine forensic casework with postmortem intervals between 6–168 h were collected. This study was approved by the Science and Ethics Committee of Xi’an Jiaotong University Health Science Center, and the methods were carried out in accordance with the approved guidelines and regulations. Informed consent was obtained from next-of-kin relatives of the cases. PMIs were certified by local police departments. The age, sex, cause of death, ambient temperature, and PMI at the time of sampling were documented for each corpse (Table 5).

**Table 5:** Basic information of each corpse used for PMI classification.

Case Name	Age (years)	Sex	Cause of Death	Place of Death	Ambient Temperature (C°)	PMI (hours)
Case A	54	Male	Coronary Heart Disease	Hospital	24	62
Case B	62	Male	Coronary Heart Disease	Hospital	24	112
Case C	25	Female	Chest Trauma	Factory	20	168
Case D	34	Female	Car Accident	Road	20	6

#### MALDI-TOF MS analysis and PMI classification

The details of the MALDI-TOF MS analysis were described in the section of MALDI-TOF MS analysis. After measurements of each collected sample, the representative data were then classified by the complete classification model of human liver tissues. The results were showed in Tables 6, 7 and 8.

**Table 6:** Real routine applications of the complete GA classification model.

Case Name	GA Classification Model			
	0 h postmortem	48 h postmortem	96 h postmortem	144 h postmortem
Case A	10.00%	83.33%	6.67%	0%
Case B	0%	11.43%	80.00%	8.57%
Case C	2.63%	5.26%	15.79%	76.32%
Case D	86.11%	11.11%	2.78%	0%

**Table 7:** Real routine applications of the complete SNN classification model.

Case Name	SNN Classification Model			
	0 h postmortem	48 h postmortem	96 h postmortem	144 h postmortem
Case A	16.67%	76.67%	3.33%	3.33%
Case B	2.86%	8.57%	82.86%	5.71%
Case C	0%	7.89%	13.16%	78.95%
Case D	80.56%	16.67%	2.78%	0%

**Table 8:** Real routine applications of the complete QC classification model.

Case Name	QC Classification Model			
	0 h postmortem	48 h postmortem	96 h postmortem	144 h postmortem
Case A	13.33%	80.00%	6.67%	0%
Case B	0%	14.29%	77.14%	8.57%
Case C	0%	7.89%	18.42%	73.68%
Case D	75.00%	19.44%	5.56%	0%

#### Effectiveness of the complete classification models

For the complete classification models, we tested whether it is possible to discriminate corpse liver tissues of different PMIs in routine forensic casework. The results showed that Case A (62 h postmortem), B (112 h postmortem), C (168 h postmortem), D (6 h postmortem) was classified for “48 h postmortem”, “96 h postmortem”, “144 h postmortem”, “0 h postmortem” with a high possibility, respectively, by all classification models. These results indicate that the classification models we have set up are useful, additional tool for the PMI estimation in routine forensic casework.

## Electronic supplementary material

Supplementary Information
