# Supplementary material for: MALDI-TOF MS as a Novel Tool for the Estimation of Postmortem Interval in Liver Tissue Samples
Source: Sci Rep. 2017 Jul 7;7:4887. doi: 10.1038/s41598-017-05216-0 (PMC5501804; doi:10.1038/s41598-017-05216-0)
Supplement: Supplementary file 1 — Supplementary Information [file 41598_2017_5216_MOESM1_ESM.pdf]

MALDI-TOF MS as a novel tool for the estimation of postmortem interval in liver tissue samples

Chengzhi Li[1,2*], Zhengdong Li[2*], Ya Tuo[3], Dong Ma[4], Yan Shi[4], Qinghua Zhang[4], Xianyi Zhuo[4], Kaifei Deng[2], Yijiu Chen[2], Zhenyuan Wang[1†], Ping Huang [2†]

Table S1. Statistics of the 57 differential peaks of various PMIs in rat liver tissues.

Mass	0 d (Average±SD)	2 d (Average±SD)	4 d (Average±SD)	6 d (Average±SD)	P
2825.5	1.74±0.55	0.91±0.33	4.11±0.52	1.97±0.36	< 0.000001
1595.48	23.52±8.08	33.74±7.26	3.36±0.9	7.21±1.55	< 0.000001
1426.77	23.26±6.99	30.24±8.51	2.83±0.69	5.73±1.24	< 0.000001
3810.22	1±0.42	0.5±0.22	2.31±0.35	1.35±0.42	< 0.000001
4621.66	0.58±0.27	0.36±0.21	1.36±0.21	0.63±0.19	< 0.000001
3905.09	0.93±0.4	0.46±0.23	2.97±0.64	1.45±0.6	< 0.000001
4890.77	0.48±0.26	0.24±0.15	1.11±0.2	0.48±0.18	< 0.000001
3186.27	1.34±0.36	0.9±0.36	3.9±0.77	1.62±0.35	< 0.000001
5041.73	0.57±0.33	0.22±0.12	1.07±0.21	0.43±0.15	< 0.000001
4968.57	2.03±1.64	0.32±0.17	1.16±0.21	0.5±0.17	< 0.000001
3630.17	0.99±0.32	0.72±0.45	2.65±0.43	3.34±2.19	< 0.000001
2718.23	1.63±0.43	0.94±0.33	5.76±1.76	1.98±0.32	< 0.000001
4052.54	0.85±0.36	0.43±0.23	2.56±0.61	1.02±0.31	< 0.000001
2588.12	1.89±0.4	1.43±0.44	9.29±2.45	2.12±0.36	< 0.000001
5079.39	0.51±0.26	0.27±0.14	1.13±0.23	0.46±0.16	< 0.000001
4748.35	0.7±0.4	0.4±0.28	1.32±0.21	0.58±0.18	< 0.000001
5162.35	0.38±0.18	0.23±0.12	1.1±0.25	0.43±0.17	< 0.000001
5304.42	0.35±0.15	0.23±0.12	0.99±0.22	0.42±0.16	< 0.000001
5386.4	0.3±0.13	0.21±0.11	1±0.24	0.37±0.15	< 0.000001
1233.12	7.77±2.37	10.03±3.15	3.04±1.2	7.46±1.87	< 0.000001
5480.28	0.29±0.13	0.21±0.12	0.87±0.21	0.35±0.14	< 0.000001
5587.12	0.28±0.14	0.19±0.1	0.85±0.22	0.35±0.16	< 0.000001
5664.89	0.28±0.14	0.18±0.1	0.82±0.22	0.29±0.11	< 0.000001
5914.26	0.24±0.12	0.15±0.09	0.74±0.21	0.25±0.1	< 0.000001
5795.67	0.26±0.12	0.17±0.09	0.86±0.25	0.29±0.11	< 0.000001
2310.6	2.26±0.43	1.68±0.4	5.58±1.7	2.4±0.33	< 0.000001
2116.01	2.38±0.5	1.63±0.44	3.15±0.58	2.8±0.36	< 0.000001
3523.49	1.06±0.36	0.76±0.43	6.05±2.26	1.93±0.89	< 0.000001
2208.15	2.71±0.39	2.05±0.38	5.5±1.66	2.47±0.4	< 0.000001
6649.07	1.33±0.97	0.27±0.33	0.55±0.23	0.16±0.06	< 0.000001
6737.09	0.16±0.1	0.1±0.07	0.44±0.16	0.14±0.06	< 0.000001

6536.86	0.16±0.09	0.13±0.09	0.52±0.19	0.16±0.06	< 0.000001
2064.29	2.54±0.53	1.76±0.45	2.82±0.5	2.75±0.38	< 0.000001
6562.44	0.19±0.1	0.13±0.09	0.56±0.23	0.16±0.06	< 0.000001
6271.26	0.89±0.63	1.08±1.1	2.41±1.38	0.33±0.19	< 0.000001
7196.34	0.1±0.05	0.08±0.05	0.39±0.17	0.11±0.05	< 0.000001
6082.29	0.31±0.17	0.22±0.14	0.9±0.39	0.29±0.12	< 0.000001
7478.44	0.09±0.04	0.07±0.04	0.28±0.13	0.1±0.04	< 0.000001
8284.79	0.05±0.02	0.05±0.03	0.15±0.06	0.09±0.06	< 0.000001
8180.83	0.07±0.04	0.07±0.04	0.19±0.08	0.6±0.75	< 0.000001
6905.81	0.16±0.08	0.16±0.09	0.43±0.18	0.14±0.06	< 0.000001
8595	0.04±0.02	0.03±0.02	0.12±0.05	0.07±0.04	< 0.000001
7001.75	0.17±0.1	0.14±0.09	0.42±0.19	0.15±0.07	< 0.000001
7690.7	0.1±0.05	0.08±0.04	0.39±0.21	0.1±0.04	< 0.000001
9654.08	0.02±0.01	0.02±0.02	0.07±0.03	0.04±0.02	< 0.000001
9543.68	0.03±0.01	0.03±0.02	0.07±0.03	0.04±0.02	< 0.000001
8017.23	0.08±0.04	0.08±0.05	0.22±0.1	0.09±0.04	< 0.000001
9939.92	0.13±0.11	0.06±0.04	0.08±0.04	0.04±0.02	< 0.000001
7096.49	0.14±0.07	0.1±0.06	0.5±0.3	0.13±0.06	< 0.000001
9795.6	0.03±0.01	0.03±0.02	0.07±0.03	0.03±0.02	< 0.000001
9244.84	0.05±0.03	0.03±0.02	0.1±0.05	0.04±0.02	< 0.000001
6363.86	0.27±0.16	0.56±0.65	1.99±1.62	0.23±0.09	< 0.000001
7931.58	0.09±0.07	0.13±0.1	0.2±0.09	0.09±0.04	< 0.000001
7599.56	0.16±0.1	0.15±0.1	0.29±0.13	0.12±0.06	< 0.000001
9045.02	0.06±0.03	0.06±0.03	0.21±0.15	0.06±0.03	< 0.000001
3461.4	2.37±1.87	4.34±4.92	3.86±1.57	2.75±1.39	< 0.000001

Table S2. Statistics of the 43 differential peaks of various PMIs in human liver tissues.

Mass	0 d (Average±SD)	2 d (Average±SD)	4 d (Average±SD)	6 d (Average±SD)	P
3335.81	3.35±0.77	4.04±0.97	1.82±0.81	0.94±0.13	< 0.000001
453.34	0.86±0.09	1.74±0.41	1.95±0.39	1.98±0.52	< 0.000001
3208.15	4.21±1.1	2.23±0.62	1.17±0.37	0.89±0.11	< 0.000001
616.57	5.28±1.32	4.31±1.35	2.11±0.74	1.73±0.35	< 0.000001
3476.85	1.3±0.22	1.52±0.4	0.96±0.15	0.88±0.11	< 0.000001
669.28	0.98±0.1	1.06±0.12	1.75±0.49	1.63±0.53	< 0.000001
1400.92	1.41±0.27	1.02±0.13	1±0.08	0.98±0.08	< 0.000001
790.99	3.59±0.87	2.31±0.75	2.61±1.36	2.64±1.72	< 0.000001
2548.3	0.86±0.08	0.86±0.08	0.97±0.11	0.92±0.12	< 0.000001
1310.34	0.94±0.11	1.08±0.15	0.98±0.08	0.97±0.09	< 0.000001
785.95	18.1±3.49	13.29±2.75	3.5±1.12	4.22±1.36	< 0.000001
1570.16	2.18±0.95	1.31±0.65	0.59±0.38	0.66±0.27	< 0.000001

5043.58	0.02±0.02	0.05±0.02	0.08±0.06	0.12±0.07	< 0.000001
1399.21	2.01±0.85	0.93±0.34	0.69±0.38	0.63±0.24	< 0.000001
656.73	3.68±1.26	5.82±2.16	6.63±2.61	7.01±1.94	< 0.000001
4679.45	0.03±0.02	0.07±0.04	0.06±0.05	0.11±0.07	< 0.000001
4732.82	0.03±0.02	0.06±0.03	0.09±0.09	0.14±0.09	< 0.000001
617.93	1.01±1.08	4.98±1.48	8.53±2.76	3.07±5.65	< 0.000001
1063.87	0.58±0.22	0.86±0.25	1.18±0.51	1.08±0.4	< 0.000001
1232.3	0.85±0.33	0.85±0.26	2.06±1.16	1.7±0.86	< 0.000001
3328.85	4.02±2.92	4.3±2.86	1.22±1.22	1.5±1.51	< 0.000001
6044.05	0.01±0.01	0.02±0.02	0.04±0.03	0.06±0.04	< 0.000001
5659.78	0.03±0.02	0.03±0.01	0.05±0.04	0.07±0.04	< 0.000001
6172.72	0.01±0.01	0.02±0.01	0.03±0.03	0.05±0.03	< 0.000001
3193.88	1.48±1.28	0.69±0.51	0.27±0.2	0.37±0.17	< 0.000001
652.44	1.99±1.46	4.42±2.56	4.2±3.46	4.64±2.56	< 0.000001
2287.78	0.17±0.08	0.24±0.1	0.29±0.18	0.36±0.19	< 0.000001
5875.22	0.02±0.02	0.03±0.02	0.05±0.05	0.08±0.06	< 0.000001
3903.34	2.28±1.84	0.41±0.45	0.28±0.28	0.35±0.22	< 0.000001
4567.53	0.38±0.42	1.26±0.95	0.32±0.45	0.74±0.69	< 0.000001
6554.71	0.01±0.01	0.01±0.01	0.03±0.04	0.04±0.03	< 0.000001
7432.28	0.31±0.38	0.02±0.02	0.04±0.04	0.05±0.03	< 0.000001
1012.11	0.62±0.3	0.87±0.25	1.18±0.76	0.97±0.4	< 0.000001
6753.36	0.01±0.01	0.02±0.01	0.03±0.03	0.05±0.05	< 0.000001
1309.34	4.34±3.04	2.04±1.17	2.99±2.73	1.63±1.13	< 0.000001
7761.92	0.07±0.09	0.01±0.01	0.03±0.03	0.04±0.03	< 0.000001
2794.53	0.38±0.34	0.45±0.32	1.13±1.14	0.77±0.54	< 0.000001
2581.53	0.85±0.74	0.34±0.17	0.39±0.29	0.55±0.32	< 0.000001
7569.95	0.04±0.02	0.03±0.02	0.05±0.05	0.07±0.05	< 0.000001
7291.83	0.01±0.01	0.01±0.01	0.02±0.02	0.03±0.03	< 0.000001
7100.88	0.02±0.01	0.01±0.01	0.03±0.03	0.04±0.05	< 0.000001
2049.74	0.27±0.2	0.24±0.09	0.33±0.2	0.4±0.29	< 0.000001
7938.93	0.02±0.01	0.02±0.01	0.03±0.03	0.04±0.04	< 0.000001

Table S3 Basic information of each case used for the establishment of PMI classification models

Case Name	Age (years)	Sex	Cause of Death	PMI 1 (hours)	PMI 2 (hours)
Case A1	52	Male	Sudden cardiac death	4	32
Case A2	67	Female	Car Accident	3	24
Case A3	49	Female	Car Accident	3	16
Case A4	72	Male	Car Accident	4	18
Case A5	64	Male	Sudden cardiac death	3	41
Case A6	57	Male	Car Accident	3	18
Case B1	28	Female	Car Accident	3	16

Case B2	66	Male	Car Accident	4	10
Case B3	78	Male	Sudden cardiac death	4	36
Case B4	42	Male	Car Accident	4	15
Case B5	21	Female	Car Accident	4	14
Case B6	83	Male	Car Accident	3	18
Case C1	78	Male	Sudden cardiac death	4	36
Case C2	76	Male	Sudden cardiac death	4	41
Case C3	61	Female	Sudden cardiac death	3	40
Case C4	42	Female	Car Accident	4	18
Case C5	58	Female	Car Accident	4	10
Case C6	68	Female	Sudden cardiac death	2	36
Case D1	55	Male	Sudden cardiac death	3	45
Case D2	72	Male	Sudden cardiac death	2	36
Case D3	66	Male	Car Accident	4	18
Case D4	56	Female	Car Accident	4	19
Case D5	86	Male	Sudden cardiac death	3	42
Case D6	64	Female	Car Accident	3	18

Case A1-6: group 1; Case B1-6: group 2; Case C1-6: group 3; Case D1-6: group 4; PMI 1 (hours): the approximate time interval between death and admission of the body at the mortuary; PMI 2 (hours): the approximate time interval between death and the autopsy
